# Hirschsprung’s Disease—Recent Understanding of Embryonic Aspects, Etiopathogenesis and Future Treatment Avenues

**DOI:** 10.3390/medicina56110611

**Published:** 2020-11-13

**Authors:** Martin Klein, Ivan Varga

**Affiliations:** Institute of Histology and Embryology, Faculty of Medicine, Comenius University in Bratislava, Spitalska Street 24, SK-813 72 Bratislava, Slovakia; ivan.varga@fmed.uniba.sk

**Keywords:** Hirschsprung’s disease, neural crest cells, neurocristopathies, etiopathogenesis, interstitial cells of Cajal, mast cells

## Abstract

Hirschsprung’s disease is a neurocristopathy, caused by defective migration, proliferation, differentiation and survival of neural crest cells, leading to gut aganglionosis. It usually manifests rapidly after birth, affecting 1 in 5000 live births around the globe. In recent decades, there has been a significant improvement in the understanding of its genetics and the association with other congenital anomalies, which share the pathomechanism of improper development of the neural crest. Apart from that, several cell populations which do not originate from the neural crest, but contribute to the development of Hirschsprung’s disease, have also been described, namely mast cells and interstitial cells of Cajal. From the diagnostic perspective, researchers also focused on “Variants of Hirschsprung’s disease”, which can mimic the clinical signs of the disease, but are in fact different entities, with distinct prognosis and treatment approaches. The treatment of Hirschsprung’s disease is usually surgical resection of the aganglionic part of the intestine, however, as many as 30–50% of patients experience persisting symptoms. Considering this fact, this review article also outlines future hopes and perspectives in Hirschsprung’s disease management, which has the potential to benefit from the advancements in the fields of cell-based therapy and tissue engineering.

## 1. Introduction

Hirschsprung’s disease (HSCR) is a developmental disorder of the enteric nervous system (ENS), caused by defective migration, proliferation, differentiation and survival of neural crest cells (NCC), leading to complete absence of ganglia in the gut wall *(aganglionosis coli)*. The Latin term for HSCR is *megacolon congenitum* [1], reflecting the typical enlargement of the intestine located orally to the affected part. Neural crest *(crista neuralis)* (NC) is an extremely important embryonic structure, which gives rise to an assorted spectrum of cell populations, including those comprising the ENS. Since the most prominent etiopathogenetic factor in the development of HSCR is the NC disorder, this condition falls into the category of neurocristopathies [2]. The resulting complete absence of ENS ganglia in the affected part of the intestine leads to functional obstruction, which clinically manifests rapidly after birth. It is the most frequent developmental disorder of the ENS, occurring once in 5000 live births around the globe, with varying regional and racial differences [3]. Gender-wise, the incidence is higher in males [4]. The most prominent early symptom is an abnormal transition of meconium, which leads to further clinical evaluation, eventually confirming the diagnosis by imaging techniques and biopsy [5]. In most cases, the inevitable therapeutic approach is the surgical resection of the aganglionic part of the intestine, however, as many as 30–50% of patients experience persisting symptoms [6]. If the symptomatology persists despite the treatment, the severity of the stasis of the gut contents can worsen, possibly leading to life-threatening Hirschsprung-associated enterocolitis [7]. The surgical management of HSCR has improved considerably over recent decades. The refinement of HSCR therapy has been directly proportional to overall advancement of paediatric surgery, so the current short-term outcomes are much more optimistic. The development of modern approaches such as single-stage trans-anal pull through has ensured milder scarring after surgery, superior pain alleviation, reduced hospitalisation time, as well as better safety and effectiveness [8]. In spite of that, a considerable proportion of patients suffer not only from the somatic facet of the persisting symptomatology, but also from various psychosocial issues related to some of the conditions persisting after the treatment, such as faecal incontinence, lowering the quality of life [9]. Another important aspect of the disease lies in its origin. As mentioned earlier, HSCR is a neurocristopathy, so the evaluation of a patient diagnosed with HSCR has to be careful and thorough in order to search for a possible occurrence of other disorders associated with the defective NC development, e.g., medullary thyroid carcinoma, neurofibromatosis or multiple endocrine neoplasia [10].

The main purpose of this review paper is to document the embryonic aspects of the HSCR with respect to other neurocristopathies and various associated diagnostic perplexities. We also review the role of other cell populations (interstitial cells of Cajal and mast cells), which do not originate from the NC, but contribute to the pathogenesis of HSCR.

## 2. Neural Crest and Neurocristopathies

NC is a temporary structure found only in vertebrates, which arises from the neural folds (*plicae neurales)* during the formation of a cylindrically shaped neural tube. Despite its neuroectodermal origin, it does not give rise exclusively to nervous tissue structures, but after undergoing epithelial-mesenchymal transition, its multipotent cells migrate to distant parts of the developing embryo and subsequently differentiate into various cell types [11]. The NC is generally subdivided into four main regions, which differ in migratory pathways and cell types, they eventually produce. We distinguish cranial (cephalic) [12], trunk [13], vagal and sacral [14,15] and cardiac NC [16]. Depending on the region, NCCs differentiate into constituents of the peripheral nervous system, including the ENS, but also to connective tissue, muscles and bones in the head and neck region, structures of the eye, ear, melanocytes, chromaffin cells of the adrenal gland and many more [17]. NCCs are not only remarkable to embryologists and evolutionary biologists. Their migratory and developmental patterns share similarities with those of malignant cells during metastasizing, so studying NC development can also provide valuable insights into cancer behaviour [18]. The importance of the NC and the vastness of different cell types it gives rise to has led some researchers to propose a reconsideration of the classic three-germ-layer model of the embryonic development, recognizing the NC as the fourth germ layer [19,20]. The first description of the NC is attributed to a Swiss anatomist Wilhelm His, Sr., who identified it in a chick embryo in the second half of the 19th century. He named the structure the intermediate cord, a translation of the original term *Zwischenstrang*. The official acceptance and inclusion of this structure into *Nomina embryologica*, under the Latin name *crista neuralis*, came almost exactly 100 years after its initial identification [21].

Robert P. Bolande, an American physician and pathologist, was the first to conceive the concept of neurocristopathies—a blanket term for a large group of disorders originating from the defective development of the NC. He also proposed the classification of these disorders into simple neurocristopathies and complex neurocristopathies/neurocristopathic syndromes based on complexity of the principal pathogenetic mechanism and the extent of the disease [22]. From a wide spectrum of different neurocristopathies, the most thoroughly understood and researched are Treacher Collins syndrome, 22q11.2 deletion syndromes and HSCR [23].

## 3. Normal Development of the Enteric Nervous System

ENS is a unique web-like system of innervation of the gut wall in the form of two plexuses located in the *tunica submucosa* (*plexus nervosus submucosus seu Meissneri*) and *tunica muscularis externa* (*plexus nervosus myentericus seu Auerbachi*). Belonging to neither the parasympathetic nor sympathetic nervous system, the ENS constitute the third distinctive subcategory of the autonomic nervous system, although it receives input from both [24]. Its complexity, autonomy and similar features to the central nervous system (CNS) has inspired researchers to portray the ENS as “the brain of the gut” or the “second brain”. To go even further, according to some authors´ evolutionary perspective, both the CNS and ENS evolved from a “primitive” ENS, postulating a theory that ENS is actually “the first brain” [25]. The exact origin of the ENS was dubious until 1954, when Yntema and Hammond demonstrated that, after ablation of vagal NC in chick embryos, they were unable to develop the enteric intramural ganglia. This observation finally established the ENS as a NC derivative [26]. Thanks to following research endeavours with more advanced approaches, using, for example, the method of quail-chick chimeric grafting, it was repeatedly confirmed that vagal NCCs are the main source of the ENS constituents [27]. There is also a population of sacral NCCs, which migrate into the distal hindgut and contribute to the ENS formation in a vagal NCCs-independent manner. As vagal NCCs are more invasive and potent during the ENS development, there have been suggestions that their transplantation into sacral neuraxis may represent a promising approach in the management of various developmental disorders affecting the hindgut, including HSCR [28]. The NCCs migration towards the gut follows a highly coordinated and structured string-like pattern with foremost cells, forming what is referred to as “wavefront” [29]. The patterns of migration and the proper settlement of NCCs in the gut are influenced by uniquely orchestrated system of interactions with different factors contributing to the whole process. One such factor is retinoic acid, secreted by somitic mesoderm and foregut endoderm adjacent to the NCCs migratory routes, which primes the migrating NCCs, so they can populate the gut wall properly [30].

## 4. Etiopathogenesis of Hirschsprung’s Disease

**Figure 1:**
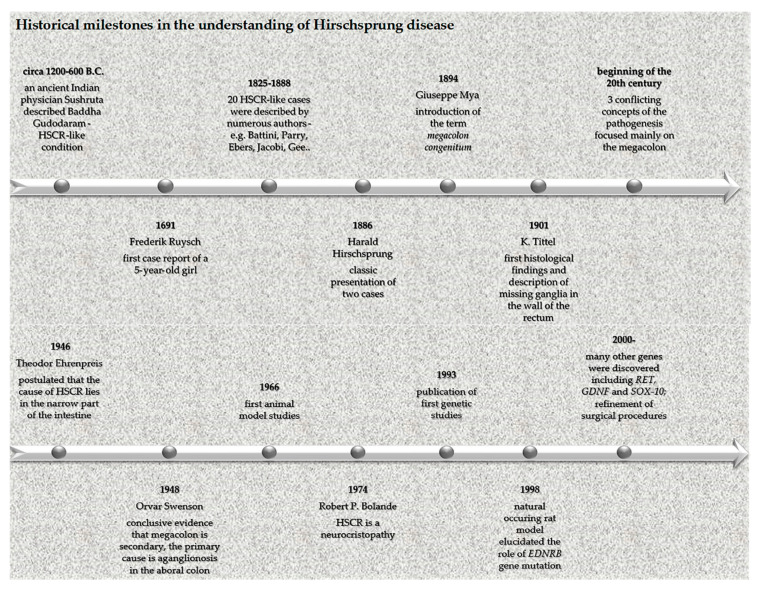
Historical milestones in the understanding of Hirschsprung’s disease.

Although this eponymous disease is named after a Danish physician Harald Hirschsprung, who described it in 1886, it was actually a Dutch anatomist and botanist, Frederik Ruysch, who first mentioned this condition in 1691 as “*enormis intestini coli dilatatio*” in a case report of a 5-year-old girl. Therefore, an eponym Ruysch disease was created, although it is rarely used, despite Ruysch´s description being almost 200 years older than Hirschsprung’s [31]. The understanding of HSCR etiopathogenesis was obscure until 1948. The reason for this is that the research focus was largely misled by the most apparent clinical sign of the condition. This classic case of cause vs. consequence misattribution directed the researchers to focus on the enlarged part of the intestine in an attempt to find the underlying pathomechanism, while the truly diseased part was neglected. A breakthrough curative rectosigmoidectomy in 1947 and subsequent surgeries and experiments in 1948 eventually revealed that the culprit was aganglionosis of the aboral part of the intestine, while the enlargement above the affected part was merely a consequence of the stasis [32]. As shown in Figure 1, the historical timeline of HSCR is very complex, with widely known, but also less renowned names contributing to its description and understanding [33,34,35].

With the advancements in molecular biology, HSCR was among the first conditions in which the genetic aspects were documented [36]. The modern study of HSCR pathophysiology employs various approaches to animal modelling. The first approach is the study of naturally occurring models. These represent a valuable possibility of researching spontaneous mutations and their effect, but are more difficult to control. For this reason, it is often necessary to surgically intervene. A good example of this scenario is an original paper by Stamp et al. (2015), in which the authors studied mice with spontaneous null mutation in endothelin receptor type B (*EDNRB*). This mutation made the mice unable to survive long enough to be suitable for experimenting. Therefore, the authors had to perform a colostomy to prolong the life of the animals so that they could be studied as models of HSCR [37]. The interventional animal models use teratogens, surgical intervention or gene knockout for the purpose of HSCR induction. These techniques have greatly advanced the understanding of the HSCR etiopathogenesis [38]. It is now established that HSCR is a heritable condition, with an estimated 10–20% of cases attributable to positive family history, while the rest account for sporadic occurrence. Given that the disease is heterogeneous, the inheritance patterns are complex and the etiology is multifactorial [39]. Multiple genes have been implicated in the failure of NCCs to effectively populate the gut and develop normally into the ENS. Some of the most thoroughly studied and significant genes whose disruptive expression contributes to the development of HSCR are *RET* and *GDNF*. *RET* is a proto-oncogene, which encodes the RET tyrosine kinase receptor. The *GDNF* gene encodes the GDNF protein (belonging to the GDNF family of ligands), which is a ligand for RET [40]. Another important protein is GFRα1. The interplay between these proteins is as follows: GFRα1 and GDNF form a complex, subsequently activating RET, which undergoes autophosphorylation, which, in turn, activates the RET pathway, leading to regulation of important embryonic tasks of enteric neural crest cells (ENCCs), including migration, survival, proliferation and differentiation [41]. A mutation in these crucial genes makes it impossible for ENCCs to migrate and further develop in precise and timely manner. In normal conditions, *GDNF* is expressed in the mesenchyme along the developing gut, which attract the *RET*- and *GFRA1*-expressing ENCCs. The *GDNF* expression then progresses caudally, ensuring the proper ENCCs colonisation of the gut [42]. Apart from these important interactions, there are numerous other genes and pathways playing a role in defective ENS development, e.g., *EDNRB*, *PHOX2B*, *SOX10* and many others [43]. The 25 most studied genes/loci according to PubMed/MEDLINE are shown in Table 1.

Although the genetics of HSCR pathogenesis is solidly understood, it elucidates roughly 50% of all cases. It follows that other factors have to be taken into consideration. One such factor is the aforementioned retinoic acid signalling [30]. Experimental approaches using mice models have demonstrated that retinoic acid is required for normal ENS formation. Fu et al. (2010) used *Rbp4−/−* mice with induced retinol-binding protein deficiency, causing mild vitamin A deficiency and mice with double mutation, *Rbp4−/−*, with additional RET signalling alteration (*Ret+/−*), which is a predisposing factor to aganglionosis. The authors found out that in case of sole RBP4 deficiency, the mice developed aganglionosis only sporadically, if their diet was rich in vitamin A. However, in the case of simultaneous RET signalling alteration, the mice developed aganglionosis even when fed a diet high in vitamin A. The disruption of these two pathways at the same time thus has a synergic action [44]. A recent paper by Uribe et al. (2018), which used a zebrafish model, also showed that retinoic acid is vital during ENCCs migration and survival [45].

## 5. Hirschsprung’s Disease and Associated Conditions

A crucial step after the diagnosis of HSCR is the further evaluation of a patient to search for additional abnormalities, as the shared pathophysiology of HSCR and other associated conditions raises the risk of their concurrent occurrence. Sláviková et al. (2015) studied a cohort of 130 paediatric patients and concluded that 26.1% of patients had associated congenital defects. When functional conditions added up to congenital disorders, more than 50% of patients had associated diagnoses. The discussed spectrum of possible HSCR-associated anomalies was truly manifold, from immune system derangements, through heart defects to genitourinary malformations [46]. Amiel et al. (2008) reviewed that, in around 70% of cases, HSCR occurs as a solitary condition, 18% of cases have other congenital anomalies and 12% of patients are diagnosed with chromosomal anomalies, with more than 90% accounting for Down syndrome. Due to these figures, the authors emphasized that the complex management of a patient diagnosed with HSCR requires thorough evaluation by an experienced dysmorphologist [47]. Apart from associated anomalies, which occur concomitantly while the HSCR is the primary pathology, HSCR has been also described as an integral part of several syndromes such as congenital central hypoventilation syndrome, some forms of Waardenburg syndrome, or Mowat-Wilson syndrome, all of which are classified as neurocristopathies. The study of HSCR in the context of these syndromes has greatly contributed to the understanding of its genetics [48]. The most frequent and well-described individual anomalies in patients with HSCR are, in descending order, gastrointestinal, CNS, genitourinary, musculoskeletal, cardiovascular, craniofacial and integumentary anomalies [49]. The range of these concomitant disorders highlights a high complexity and diversity of NCCs and their hugely significant role during the normal development of many, seemingly unrelated, organ systems.

Apart from morphological anomalies, HSCR is also associated with various functional disorders. Despite their significance, the number of papers discussing them is scarce. To the best of our knowledge, there is only one paper authored in 1988 by Kushch et al. outlining the association between congenital anomalies of the large intestine and defective development of the immune system [50]. This is unexpected, considering that the normal development of the thymus, the organ that is paramount to the proper development of adaptive cellular immunity, depends on the interaction between developing epithelial thymic primordium and surrounding mesenchyme. This NC-derived mesenchyme originates from the same NC region, which also gives rise to future intestinal ganglia [51]. The early thymic organogenesis is not the only stage of its development with an important role of NC-derived cells. Later in the thymus development, they differentiate into perivascular cells and possibly contribute to such important functions as the formation of the thymus-blood barrier and regulation of the endothelial function [52].

Another under-researched, HSCR-associated functional anomaly is congenital hypothyroidism. This is also surprising, since it is a well-established fact that thyroid hormones are vital in proper development of the cerebral cortex [53]. In 35 years, which have passed from the first description of a possible connection between HSCR and congenital hypothyroidism in 1985 [54], only five studies have been published on the topic, two of which are case reports [46,55,56,57,58]. From the embryonic perspective, the anomalous development of the thyroid gland can also be considered a neurocristopathy, since NCCs play an important role in the development of connective tissue components of the gland and its calcitonin-producing parafollicular cells [46,59].

## 6. Interstitial Cells of Cajal and Hirschsprung’s Disease

Interstitial cells of Cajal *(cellula enterica interstitialis stimulans)* (ICCs) are pacemaker cells, vital for the normal coordination of gastrointestinal motility. Their embryonic origin is distinct from that of the ENS, although they were first described as “interstitial neurons” by their discoverer Santiago Ramón y Cajal at the end of the 19th century. They are derivatives of c-kit positive precursor cells of mesenchymal origin [60]. From among a variety of functions of ICCs with regard to gut motility, their action as transducers of neural impulses from the ENS towards the smooth muscle is worth mentioning. They also play a pivotal role in the generation of slow waves of electrical activity in smooth muscle cells, which is important for proper rhythmic orchestration of peristaltic movements [61]. During the organogenesis of the gut, NCCs directly influence the differentiation of mesenchymal stem cells into c-kit precursors of ICCs, while, at the same time, these newly differentiated cells reciprocally influence NCCs in several ways. They possibly have a regulatory role in NCCs migration via the reduction in GDNF, pointing the migratory wave of NCCs in the right direction. They probably also induce NCCs differentiation into mature ENS constituents [62]. As far as HSCR is concerned, there is a high probability that the disturbances of ICCs are co-responsible for its development. Morphological studies detailing the distribution of ICCs in patients with HSCR were published back in the 1990s and early 2000s. Vanderwinden et al. (1996) demonstrated a reduced cellular density and disruption of ICCs network in patients with HSCR [63]. Rolle et al. (2002) performed a morphological study to evaluate the distribution of ICCs. They found out that ICCs were significantly reduced in the specimens from HSCR patients. They also suggested that the ICCs alteration might be responsible for persisting dysmotility after surgery [64]. On the other hand, Newman et al. (2003) found no alteration in ICCs in HSCR patients, nor did they find ICCs to be responsible for post-operative dysmotility [65]. A more recent experimental study used an aganglionic rat model, in which the researchers performed a simultaneous transplantation of neuroepithelial stem cells and ICCs into the aganglionic gut. The resulting differentiation of ENS components was sped up compared to the exclusive transplantation of the stem cells [66].

## 7. Mast Cells and Hirschsprung’s Disease

Mast cells (MCs) are immune cells, which carry out their main roles in a vast array of different peripheral tissues, where they migrate from the bone marrow. The most striking clinically relevant role of MCs is their involvement in allergic reactions, but they also contribute to normal bodily functions such as wound healing, angiogenesis and immune tolerance [67]. With respect to the gastrointestinal tract, MCs play a regulatory role in many crucial gut functions, e.g., epithelial permeability/epithelial barrier integrity, neuroimmune reactions, blood flow and peristaltic movements [68]. Only a handful of papers focused on the role of MCs in the pathogenesis of HSCR. In 1999, Kobayashi et al. authored an experimental study where they used immunohistochemistry to assess the number of MCs in the ganglionic, transitional and aganglionic segments of the large intestine in patients with HSCR. The aganglionic segment contained a higher quantity of MCs in all layers compared to the other two studied segments. The close contact of MCs with nerve fibres, their possible physiological role in nerve fibres growth and repair and their secretion of nerve growth factor suggest that MCs might be responsible for nerve trunk hypertrophy and hyperplasia of adrenergic and cholinergic nerve fibres—typical signs of HSCR [69]. Similar results were reproduced by Demirbilek et al. [70]. Hermanowicz et al. also focused on the size of MCs and concluded that MCs were significantly enlarged in both *t. submucosa* and *t. muscularis externa* in patients with HSCR [71]. Nevertheless, the precise role of MCs in the HSCR pathogenesis in not yet understood [67].

## 8. Diagnostic Dilemmas Associated with Hirschsprung’s Disease Evaluation

Nowadays, the establishing of HSCR *per se* is not a diagnostic perplexity. After the onset of the first signs and symptoms, a patient undergoes anorectal manometry and imaging examination, most commonly via plain abdominopelvic skiagram and contrast enema. The definitive diagnosis is confirmed by histopathologic examination, which is considered a gold standard, with 93–98% sensitivity and specificity [72]. Apart from the aganglionosis, the most typical histopathological traits of the disease are hypertrophy of nerve fibre bundles and amplified histochemical staining for acetylcholinesterase and irregular nerve fibers in *lamina propria* and *lamina muscularis mucosae* [73]. The increased activity of acetylcholinesterase is considered pathognomonic for HSCR [74]. A diagnostic dilemma can occur when a patient´s clinical presentation is similar to HSCR, however, the biopsy evaluation shows the presence of ganglia. This category of conditions is quite heterogeneous and comprises disease entities such as intestinal neuronal dysplasia, intestinal ganglioneuromatosis, isolated hypoganglionosis, immature ganglia and others [75]. The umbrella term for such conditions is non-consensual. These “variants of Hirschsprung disease” are usually referred to as chronic idiopathic intestinal pseudoobstruction, pseudo-Hirschsprung disease, neonatal intestinal pseudoobstruction, or intestinal hypoperistalsis syndrome [76]. The term “variants of Hirschsprung disease” is problematic from two points of view. First is that “Hirschsprung” automatically evokes aganglionosis, while the main feature of these conditions is that there is an incomplete absence of ENS ganglia. Secondly, the pathogenesis of some of these entities is distinct and more closely related to other syndromes. Thus, it has also been suggested to classify these conditions into a category of “Variant Enteric Nervous System” [73].

Although the diagnosis of both HSCR and pseudo-HSCR is common during early childhood, there are several case reports in the literature which describe patients who had not been properly diagnosed until adulthood. Ito et al. authored a case report of a young man (20 years of age), with a history of constipation since the age of 2, who was admitted to the hospital for cardiac arrest and died the following day. His condition was revealed during autopsy—hypoganglionosis with megacolon—while the direct cause of death was severe intestinal necrosis [77]. The ENS, especially that located in the colon, undergoes age-related morphological and functional changes, so diagnosing a gut dysmotility in aged individuals is more intricate [78,79].

## 9. Conclusions and Future Perspectives

From the embryological perspective, HSCR is far better understood currently compared to understanding several decades ago; researchers have also elucidated the multifaceted etiopathogenesis of the disease in terms of genetic interplay. However, the most challenging aspect of the disease is still its treatment. Surgical management has also significantly improved over the years, with better outcomes for a patient, but still there are many postoperative complications which can, in some cases, decrease a patient´s quality of life. Therefore, highly promising future prospects are focused on stem cell research and tissue engineering. In 2016, Rollo et al. performed an experiment, which attempted to demonstrate three possible scenarios: 1. the potential for human ENS-derived cells, obtained postnatally, to colonize an embryonic intestine, 2. the capacity of a postnatally obtained aganglionic portion of the colonic muscle from a HSCR patient to nurture the formation of ENS using murine-derived embryonic ENS cells, 3. the ability of ENS cells obtained from the ganglionic portion of a HSCR patient´s gut to autologously colonize the aganglionic part of the same patient´s colonic muscle. All three scenarios proved to be viable. The most important conclusion was that the autologous transplantation is possible thanks to the fulfilment of the most important criteria: the possibility of harvesting of ENS-derived cells from a HSCR patient, the capacity of aganglionic muscle to accept and support ENS de novo establishment, and finally, yet importantly, the ability and competence of postnatal ENS-derived cells to colonize the colonic muscle of the same patient. These results are promising, but still far from routine clinical application. The principal challenge is to generate a sufficient quantity of progenitor cells and to find adequate means to effectively transplant them to a significantly large area of the aganglionic gut [80]. In 2017, Schlieve et al. examined the potential of ENCCs derived from human pluripotent stem cells to establish a neuronal network in a tissue-engineered small intestine derived from human intestinal organoids. The results have also shown that tissue engineering and stem cell therapy is a hopeful future approach to the treatment of gut neuropathies [81]. A similar methodology was implemented in the same year by Workman et al., who used embryonic and induced pluripotent stem cells (iPSCs) to produce human gut tissue with properly functioning ENS [82]. Nowadays, cell therapy using iPSCs is a promising approach in regenerative medicine, so perhaps the knowledge obtained from their study in other diseases could also be widely applied [83]. Another promising avenue of modern treatment is genetic manipulation. A state-of-the-art CRISPR/CAS9 technology has been tested as a tool to repair RET mutations in ENCCs, responsible for their defective migration and differentiation [84]. All these novel techniques of HSCR therapy are conceivable as highly promising alternatives or complements to surgical management, nevertheless, plenty of future experimental work in this area is necessary before these preliminary optimistic results can be successfully applied to clinical practice.

## Figures and Tables

**Table 1:** The 25 genes and/or loci, which have been studied most in Hirschsprung’s disease (entries according to PubMed/MEDLINE).

Gene/Locus	PubMed/MEDLINE Entries
*RET*	597
*EDNRB*	236
*GDNF*	172
*SOX10*	144
*PHOX2B*	98
*EDN3*	87
*ZFHX1B*	43
*RMRP*	37
*NRG1*	29
*GFRA1*	26
*L1CAM*	25
*SHH*	16
*ECE1*	15
*NRTN*	10
*KIAA1279*	9
*SEMA3D*	6
SEMA3C	5
*Gli1*	5
*9q31*	4
*DHCR7*	3
*3p21*	3
*PSPN*	2
*NTF3*	2
*NTRK*	2
*19q12*	2
